# Prescribing to protect case series: naltrexone as a novel intervention to prevent fentanyl overdose in people who use cocaine

**DOI:** 10.1186/s13722-026-00701-2

**Published:** 2026-07-22

**Authors:** Rachel Xue, Justin Alves, Marielle Baldwin, Jiarui Zhang, Colleen Labelle, Alexander Y. Walley

**Affiliations:** 1 Department of Family Medicine, Boston Medical Center, 801 Massachusetts Avenue Boston, Boston, MA 02118 USA; 2 Department of Medicine, Section of General Internal Medicine, Boston University Chobanian & Avedisian School of Medicine and Boston Medical Center, Boston, MA USA

**Keywords:** Naltrexone, Stimulant use disorder, Cocaine-related disorders, Harm reduction, Fentanyl

## Abstract

**Background:**

The rise in fentanyl contamination of cocaine has contributed to a surge in opioid-involved overdose deaths among people who use cocaine (PWUC). Existing harm reduction strategies, including naloxone distribution, drug checking, and counseling people to use with others, are inadequate in fully addressing this risk. Despite robust evidence supporting contingency management (CM) as an effective treatment for cocaine use disorder (CUD), its implementation remains limited. Naltrexone, a potent opioid antagonist, has been evaluated for treatment of CUD and has not been found to be effective. No pharmacotherapies are FDA approved for CUD. However, naltrexone may provide opioid overdose protection for individuals at risk of unwitting fentanyl exposure through fentanyl contamination of cocaine.

**Case presentation:**

We conducted a retrospective case series of three adults with cocaine use disorder receiving care at the Stimulant Treatment and Recovery Team (START) Clinic at Boston Medical Center. All three patients with cocaine use disorder reported cocaine use with unintentional fentanyl exposure, and two had prior nonfatal opioid-involved overdoses. Oral or intramuscular naltrexone was initiated in all cases and was tolerated without reported adverse effects or precipitated withdrawal. Patients continued naltrexone for several months, and no opioid-involved overdoses were reported.

**Conclusions:**

In this case series of patients with cocaine use disorder, the initiation of naltrexone for opioid overdose risk prevention was feasible, acceptable, and well tolerated. Naltrexone warrants study as a component of overdose prevention strategies in stimulant treatment settings where fentanyl contamination poses increased risk.

## Background

Unregulated fentanyl in the drug supply that has resulted in the deaths of thousands of people who use drugs (PWUD) annually [20]. In Massachusetts, the percentage of overdose fatalities with fentanyl present surged from less than 40% in 2014 to greater than 90% from 2019 to 2024. An unknown but increasing number of opioid-involved overdose deaths have been occurring among people who use stimulants, specifically those who use cocaine and methamphetamine [19]. Emergency department data and community drug checking consistently show widespread fentanyl contamination of stimulant supplies, resulting in unintended fentanyl exposure and overdose risk, particularly among Black and Hispanic populations [2, 3, 6, 25].

Nationally, cocaine and methamphetamine are the most commonly used unregulated stimulants [22] and increasingly present with fentanyl in the toxicology of people who die from overdose [8, 14, 26]. The surge in overdose deaths that involve fentanyl and cocaine, or methamphetamine has included people with intentional polysubstance use and people with unintentional use of fentanyl-contaminated stimulants. Thus, opioid overdose prevention efforts should explicitly include people who use cocaine and methamphetamine. Core overdose safety practices should be adapted for people using cocaine and methamphetamine and include access to naloxone and training in its use, overdose recognition and response planning, drug checking and fentanyl test strips where available, avoidance of polysubstance use, use in the presence of others capable of responding, cautious dosing to assess potency, and heightened vigilance among individuals without opioid tolerance, including many people who primarily use cocaine or methamphetamine [18, 21]. Medications such as naltrexone may mitigate opioid-involved overdose risk and thus warrant further exploration.

Naltrexone is a potent opioid antagonist approved for the treatment of opioid and alcohol use disorders and has demonstrated the ability to block the effects of opioids, including fentanyl, and reduce craving for and use of alcohol and opioids [12, 23]. A randomized controlled trial among treatment-seeking people with methamphetamine use disorder demonstrated significant, though modest, increased methamphetamine abstinence for 380 mg of naltrexone injected every 3 weeks in combination with 450 mg of daily oral buproprion [24]. Multiple randomized trials evaluating naltrexone alone and in combination have consistently demonstrated no significant reduction in cocaine use, self-administration, or sustained abstinence [4, 17]. However, the failure of naltrexone to reduce cocaine use does not preclude its potential utility as an overdose prevention strategy in the context of a fentanyl-contaminated cocaine supply. When prescribed to maintain sufficient plasma concentrations through oral or extended-release formulations, naltrexone provides sustained mu-opioid receptor blockade and will block the effects of inadvertent fentanyl exposure and reduce risk of opioid overdose [12]. A trial protocol for this overdose prevention strategy was published in early 2026 [1]. Otherwise, there are no peer reviewed reports of naltrexone for opioid overdose protection among people using cocaine that we are aware of. Naltrexone is FDA-approved for the treatment of alcohol and opioid use disorders, but not specifically for the prevention of opioid overdose. We present a case series of three patients with cocaine use disorder who were treated with oral or injectable naltrexone for the purpose of reducing the overdose risk from unintentional use of fentanyl.

The three patients in this retrospective case series received care at the Stimulant Treatment and Recovery Team (START) Clinic, an outpatient stimulant treatment program at Boston Medical Center. We identified the cases between October 2023 and January 2026 through clinical records of patients with cocaine use disorder who reported cocaine use and unintentional fentanyl exposure and were considered at increased risk for opioid-involved overdose. Patients with primary methamphetamine use disorder were not included in this case series, as clinical practice involved offering intramuscular naltrexone administered every 21 days in combination with high-dose bupropion as a primary treatment for methamphetamine use disorder. Oral or intramuscular naltrexone was prescribed to patients included in this series. When extended-release intramuscular naltrexone was used for overdose prevention among patients with cocaine use disorder, it was administered every 4 weeks (28 days). The decision to initiate naltrexone, formulation selection, and dosing were determined by treating clinicians based on individual clinical assessment. Prior to naltrexone initiation, patients underwent clinical assessment for recent opioid use, risk of precipitated withdrawal and urine toxicology testing was obtained when clinically indicated. Patients were identified by clinic providers. Clinical data were extracted from electronic medical records using a data extraction guide generated by the authors. Written informed consent for inclusion and publication of clinical details was obtained from all participants. Per institutional guidance, IRB review was not required for this case report.

## Case presentation

Patients with cocaine use disorder treated at the START program, including the three patients included in this case series, routinely received harm reduction supplies, including safer smoking kits, fentanyl test strips, naloxone, referral to local drug checking services and counseling, including encouragement to use with other people equipped to respond, to check their drugs when possible, and to start low and go slow. All patients were enrolled in the START clinic’s contingency management program. Naltrexone was covered by MassHealth insurance for all patients, and no out-of-pocket costs were reported.

Case 1 is a 53-year-old cisgender male who presented to the clinic in 2023 with a primary diagnosis of cocaine use disorder and a history of cocaine use since age 25. He sought treatment following unintentional fentanyl exposure and non-fatal opioid-involved overdose while using cocaine. His overdose was reversed with community naloxone. During his intake visit, a urine toxicology screen was negative for opioids and positive for cocaine. The patient reported ongoing cocaine use and co-occurring alcohol use disorder and was prescribed oral naltrexone to address both his alcohol use and as a preventive measure against a potential overdose from fentanyl-contaminated cocaine. He was additionally prescribed topiramate to treat his cocaine use disorder. In 2024, this patient transitioned to intramuscular (IM) naltrexone given difficulties with adherence to oral naltrexone. He tolerated both medications well and experienced no side effects, overdoses, or opioid-related symptoms. He also reported a reduction in alcohol use, with naltrexone identified as a protective factor in his treatment continued to receive monthly naltrexone injections in January 2026.

Case 2 is a 57-year-old cisgender female who presented to the clinic in 2022 with a primary diagnosis of severe cocaine use disorder and a history of cocaine use since age 18. Her engagement in the program included weekly behavioral health and medical group therapy sessions. She continued to use cocaine one to two times per week, with brief and intermittent periods of remission. Prior to initiation, urine toxicology was negative for opioids. In 2024, intramuscular (IM) naltrexone was initiated for overdose protection. She tolerated the medication well without adverse effects, overdoses, or opioid-related symptoms. Through January 2026, she remained adherent to the monthly IM naltrexone regimen and experienced her longest period of cocaine abstinence, exceeding twelve months.

Case 3 is a 77-year-old cisgender male who presented to the clinic in 2023 with a primary diagnosis of cocaine use disorder. His substance use history started with exposure to alcohol at age 6 and exposure to cocaine at age 30. His engagement in the program included regular attendance in behavioral health, medical, and art therapy groups. At intake, he reported smoking cocaine and drinking alcohol together five to six days per week. He reported three lifetime overdoses from opioid-contaminated cocaine, the most recent of which occurred two years prior to his engagement in stimulant specific treatment and was managed by a responding bystander. Given his alcohol use disorder, he had been prescribed oral naltrexone since 2014. However, due to reported non-adherence and advancing age, he was transitioned to intramuscular (IM) naltrexone for improved adherence and overdose prevention. After transitioning to IM naltrexone, the patient came regularly to his injection visits, tolerated the medication well without adverse events and without opioid-related symptoms or additional overdoses. To further optimize treatment of his cocaine use disorder and ongoing cocaine use, topiramate was added. He remained engaged in ongoing treatment through 2025 and was enrolled in the Contingency Management Program.

## Discussion

In the three cases presented above, each presented to a primary care-based stimulant use disorder program with cocaine use disorder, and two had prior overdose from fentanyl-contaminated cocaine. Following initiation of oral or intramuscular (IM) naltrexone, patients remained engaged in care over follow-up and experienced no opioid-involved overdoses.

There were initial concerns among patients and providers that initiating naltrexone in patients potentially exposed to low levels of opioids through contaminated stimulants could precipitate withdrawal, given its opioid antagonist properties. Extended-release intramuscular naltrexone is generally well tolerated but is associated with known adverse effects, most commonly nausea, headache, and injection site reactions. However, in all cases described, patients tolerated both oral and intramuscular naltrexone without experiencing precipitated withdrawal or clinically significant adverse effects, while all 3 cases transitioned to IM naltrexone due to challenges with oral adherence.

Although naltrexone may offer potential benefit against fentanyl contamination in individuals with CUD, it represents one component of a broader overdose prevention approach. Many strategies are being considered as a way to address the lethal drug supply including expanding access to substance use treatment, harm reduction services that include drug checking with point of care test strips and mass spectroscopy molecular identification, or other substances regulated by the government and free of contamination, safe consumption sites and overdose prevention centers, and increased access to naloxone for all people [5, 7, 9]. While naltrexone is a valuable tool, it should be used in conjunction with other harm reduction strategies to effectively reduce overdose deaths. In cases 1–3, patients experienced nonfatal opioid-involved overdoses prior to initiating naltrexone for protection despite access to drug checking services and overdose education with naloxone distribution (OEND), highlighting the need to explore complementary strategies to address overdose risk in this population.

Naltrexone is a potent antagonist with a high affinity for the mu-opioid receptor which contributes to implementation challenges for treatment of OUD but may be useful and feasible in patients with co-occurring alcohol and cocaine use disorders in settings where fentanyl contamination is prevalent. In cases 1, and 3 patients had a co-occurring alcohol use disorder. In Case 1, reductions in alcohol and cocaine use were reported following initiation of naltrexone and topiramate. Given the concurrent use of topiramate, the specific contribution of naltrexone to changes in cocaine use cannot be determined. Consistent with the current literature, the appropriate use of medication for alcohol use disorder to target patients who co-use alcohol and cocaine may be an effective strategy to reduce overall cocaine consumption, a finding that aligns with the real-world implementation of this approach [16]. Importantly, in cases 1 and 2 these patients did not identify alcohol reduction as their primary treatment goal but were motivated to prevent a fatal overdose from accidental fentanyl contamination. This may represent an unexplored strategy to improve the uptake and adherence of MAUD in patients with a co-occurring stimulant use disorder.

In cases 1 and 3, patients transitioned from daily oral naltrexone to monthly injectable naltrexone due to adherence challenges with daily oral medication and not because of side effects. This observation highlights the importance of shared decision-making when selecting naltrexone formulation, considering patient preferences, lifestyle, and ability to adhere to oral medication. Patient-centered approaches, which emphasize individualized care and autonomy, have been shown to improve adherence and treatment outcomes in substance use disorder management [13]. Consistent with existing literature on preventive therapies, such as oral contraception and HIV pre-exposure prophylaxis (PrEP), long-acting formulations may support sustained engagement when daily adherence is difficult [11]. A tailored approach that accounts for individual preferences along with the ongoing risk of overdose is crucial if we are to maximize naltrexone’s potential role in overdose risk mitigation. This patient-centered approach should include partnering with patients to decide the duration of naltrexone treatment.

In cases 1 and 3, patients had documented histories of non-fatal overdoses from contaminated cocaine and were therefore amenable to the concept of pre-exposure prophylaxis for opioid overdose. This aligns with evidence that individuals with primary StUD may underestimate their risk of opioid overdose until they are experiencing one firsthand [10, 15]. Public health messaging should include PWUSt as part of a comprehensive overdose prevention strategy and to improve risk awareness and overdose preventive interventions. Notably, this case series does not include three patients engaged in treatment at the stimulant clinic who were offered and declined naltrexone as a prescribe-to-protect strategy and subsequently died from fatal opioid-involved overdoses. These overdose deaths highlight the danger faced by people who use cocaine in the setting of fentanyl contamination and underscore the promise for prospective investigation of naltrexone as an overdose prevention strategy for PWUSt.

For three patients in the START clinic with cocaine use disorder, prescribing naltrexone to protect them from overdose from fentanyl-contaminated cocaine was feasible and acceptable. We recommend providers consider this approach in similar patients. While these cases provide anecdotal evidence, further research is warranted to refine naltrexone guidelines, dosing, and long-term safety in this specific context. Overcoming barriers such as cost, limited availability of naltrexone in certain geographic areas, stigma surrounding treatment, lack of healthcare provider training, and inadequate insurance coverage, especially in marginalized communities, is crucial to ensuring equitable access to and use of this intervention.

## Conclusions

Further research is needed to establish broader acceptability and efficacy of naltrexone as a viable pharmacologic option for mitigating opioid-involved overdose risk in PWUSt. Larger, prospective studies are needed to evaluate its effectiveness, optimal implementation, and potential role alongside established harm reduction, particularly among populations disproportionately affected by overdose. Future studies should explore how naltrexone can complement existing harm reduction strategies, such as naloxone distribution, within a comprehensive overdose prevention framework. As the overdose crisis evolves, a rigorous, equity-centered research agenda is essential to determine how best to leverage naltrexone to protect those at highest risk of death.

## Data Availability

The datasets generated and/or analyzed during the current study are not publicly available due to the inclusion of sensitive, potentially identifiable clinical information, but are available from the corresponding author on reasonable request.
